# Lessons from health insurance responses in counteracting COVID-19: a qualitative comparative analysis of South Korea and three influential countries

**DOI:** 10.1186/s13690-023-01209-w

**Published:** 2023-11-21

**Authors:** Hey Jin Ko, Eunji Yun, Boryung Ahn, Hyejin Lee, Won Mo Jang, Jin Yong Lee

**Affiliations:** 1 Human Resource Administration Department, Health Insurance Review & Assessment Service, Wonju-Si, Gangwon-Do Republic of Korea; 2 Division of Pharmaceutical Policy Research, HIRA Research Institute, Health Insurance Review & Assessment Service, Wonju-Si, Gangwon-Do Republic of Korea; 3 Division of Review and Assessment Research, HIRA Research Institute, Health Insurance Review & Assessment Service, Wonju-Si, Gangwon-Do Republic of Korea; 4 Department of Family Medicine, Seoul National University Bundang Hospital, Bundang-Gu, Seongnam-Si, Gyeomggi-Do Republic of Korea; 5 Department of Family Medicine, Seoul National University College of Medicine, Jongno Gu, Seoul, Republic of Korea; 6 Department of Public Health and Community Medicine, Seoul Metropolitan Government-Seoul National University Boramae Medical Center, 20 Boramae-Ro 5-Gil, Dongjak-Gu, Seoul, 07061 Republic of Korea; 7 Department of Health Policy and Management, Seoul National University College of Medicine, 101 Daehak-Ro, Jongno-Gu, Seoul, 03080 Korea; 8 Public Health Care Center, Seoul National University Hospital, Jongno-Gu, Seoul, Republic of Korea; 9 HIRA Research Institute, Health Insurance Review and Assessment Service, Wonju-Si, Gangwon-Do Republic of Korea

**Keywords:** Pandemic, COVID-19, Health insurance, Surge capacity

## Abstract

**Background:**

The COVID-19 pandemic has caused delays and restrictions in providing medical services. In response to the medical surge, countries with social insurance systems provided financial incentives to medical institutions. This study aimed to present the directions for health insurance support by comparing countries in terms of the domains and contents of COVID-19 health insurance support to ensure timely support in case of future pandemics.

**Methods:**

An analysis framework was developed to compare health insurance policy interventions for COVID-19 and non-COVID-19 domains, and detailed policy interventions were divided into sub-domains (space, staff, and stuff) for each domain. Data were collected by country from the websites of the Ministry of Health and Social Insurers, Organisation for Economic Co-operation and Development, and European Observatory on Health Systems and Policies and were analyzed using qualitative comparative analysis.

**Results:**

The countries provided comprehensive support for both the COVID-19 and non-COVID-19 domains. In the COVID-19 domain, overall support was provided in all three sub-domains. Additional cost support was provided to prevent infection and provide secure facilities to treat confirmed patients. Outpatient services were mainly supported, and an intensive intervention was developed in the staff sub-domain for the non-COVID-19 domain. The point of policy intervention was the surge of the first confirmed case. Continuous revisions were subsequently made. The government provided financial support through health insurance.

**Conclusions:**

Regarding where policy support through health insurance should be focused, the workload of medical personnel increased according to the change in the service provision environment due to the pandemic, and the medical service delivery system changed to prevent further infection. Consequently, incentives should be provided to aid the provision of stable services to patients and should be an auxiliary means to implement the national quarantine policy more effectively via a health insurance response system that promptly provides additional financial support in case of future crises.

**Supplementary Information:**

The online version contains supplementary material available at 10.1186/s13690-023-01209-w.

**Table:** 

Text box 1. Contributions to the literature	
• Most discussions on COVID-19 have been from the perspective of the overall health system, including epidemic prevention policy, health system governance, and health service delivery • When it comes to health insurance, while there are some studies on payment systems, a comprehensive review of the intervention areas and contents is lacking • Therefore, this study compares the domains and contents of health insurance policy interventions applied to healthcare service providers in the three countries that have had a significant influence on Korea and the Korean health insurance system to present directions for establishing future response strategies	

## Background

The outbreak of coronavirus disease 2019 (COVID-19), the third pandemic declared by the World Health Organization, has led to changes in global healthcare services. COVID-19 is highly contagious and prone to asymptomatic transmission, leading to a rapid increase in infections and a consequent shortage of hospital beds and protective supplies in medical institutions. As a result, a burden was placed on service provision by medical institutions, which included obtaining temporary beds and reallocating existing health care practitioners’ responsibilities to treat COVID-19 patients. The situation has since escalated into a public health crisis due to delays and limitations in the provision of existing medical services (e.g., primary medical services for patients other than those infected with COVID-19) [1].

The surge in demand for health services during a pandemic makes it difficult to respond using the existing emergency medical system [2]. A strategy is thus required to strengthen the healthcare system’s resilience. The key inputs of the six building blocks for strengthening the healthcare system (service delivery, health workforce, health information systems, access to essential medicines, financing, and leadership/governance) involve financing and the health workforce [3, 4].

For resilience, the most important of these inputs is the financing mechanism [5]. The main financial resources in the healthcare systems of countries that operate social health insurance are taxes and contributions. The roles and importance of such financing may vary depending on the phase of the disaster. In terms of the preparedness for a pandemic, preparedness can be improved comprehensively and uniformly by using tax funding; and during the stages of disaster response, both taxes and contributions must be coordinated [6].

To address COVID-19, health insurance systems implemented additional strategies, such as altering their reimbursement systems or additionally reimbursing COVID-19-related claims [7]. In general, tax funding was provided to support these measures. In Germany, the government provided a tax subsidy when expenditures increased because of COVID-19 to address the shortfall in its social health insurance funds [8]. South Korea (hereafter “Korea”) also provided financial support through its health insurance system in addition to compensation from the government for medical institution losses in the form of supplier incentives, such as securing and distributing funds to respond to the pandemic [9].

Many countries, including Korea, have actively leveraged their health insurance systems to ensure surge capacity. However, in Korea, during the pandemic, there were instances of confusion among policymakers and healthcare institutions because of the absence of concrete discussions regarding the decision-making processes, methods, scope, and scale of health insurance policies in public health crises. The health insurance system of Korea is based on the National Health Insurance (NHI) system, which is an integrated, single-insurer system [10]. The implementation and reform of this system was influenced by countries with social insurance. Among the countries that operate a public health insurance system, Japan, Germany, and the United States have often been mentioned in previous studies as comparisons for the Korean health insurance system [10–12].

Additionally, the importance of financial support through social insurance has been increasingly emphasized in building a stable financial procurement system with frequent exposure to crises [8]. However, most discussions about COVID-19 were mainly held from the perspective of the overall health system, including epidemic prevention policy, health system governance, and health service delivery [8, 13, 14]. When it comes to health insurance, there are some studies on payment systems [15, 16]; however, a comprehensive review of the intervention areas and contents is lacking.

Therefore, this study aimed to compare and analyze the domains and contents of health insurance policy interventions applied to healthcare service providers in the three countries that have had a significant influence on Korea and the Korean health insurance system to present the directions for health insurance support for establishing response strategies for future pandemics.

## Methods

### Study design and setting

This study comparatively analyzed the insurance domains of four countries (Korea, Japan, Germany, and the United States of America) and the detailed methods and support levels in the first year (January–December 2020) of the COVID-19 pandemic. The analyzed countries, including Korea, are nations that have implemented public health systems. These countries have influenced Korea’s health insurance system reform and development and have similar economic power as members of the Organisation for Economic Co-operation and Development (OECD). Furthermore, these are countries that have actively responded to COVID-19 through their health insurance systems. To examine and analyze each country’s health insurance policies implemented in response to COVID-19, this study employed a case-based approach—qualitative comparative analysis (QCA). QCA is a case-oriented research method that can produce empirically well-grounded and context-sensitive evidence that is useful in decision-making, implementation, and evaluation [17, 18]. This methodology is not only widely used in healthcare policy studies [19–21] but is also being applied in studies on the provision of healthcare services during the COVID-19 pandemic [14, 22].

**Fig. 1:**
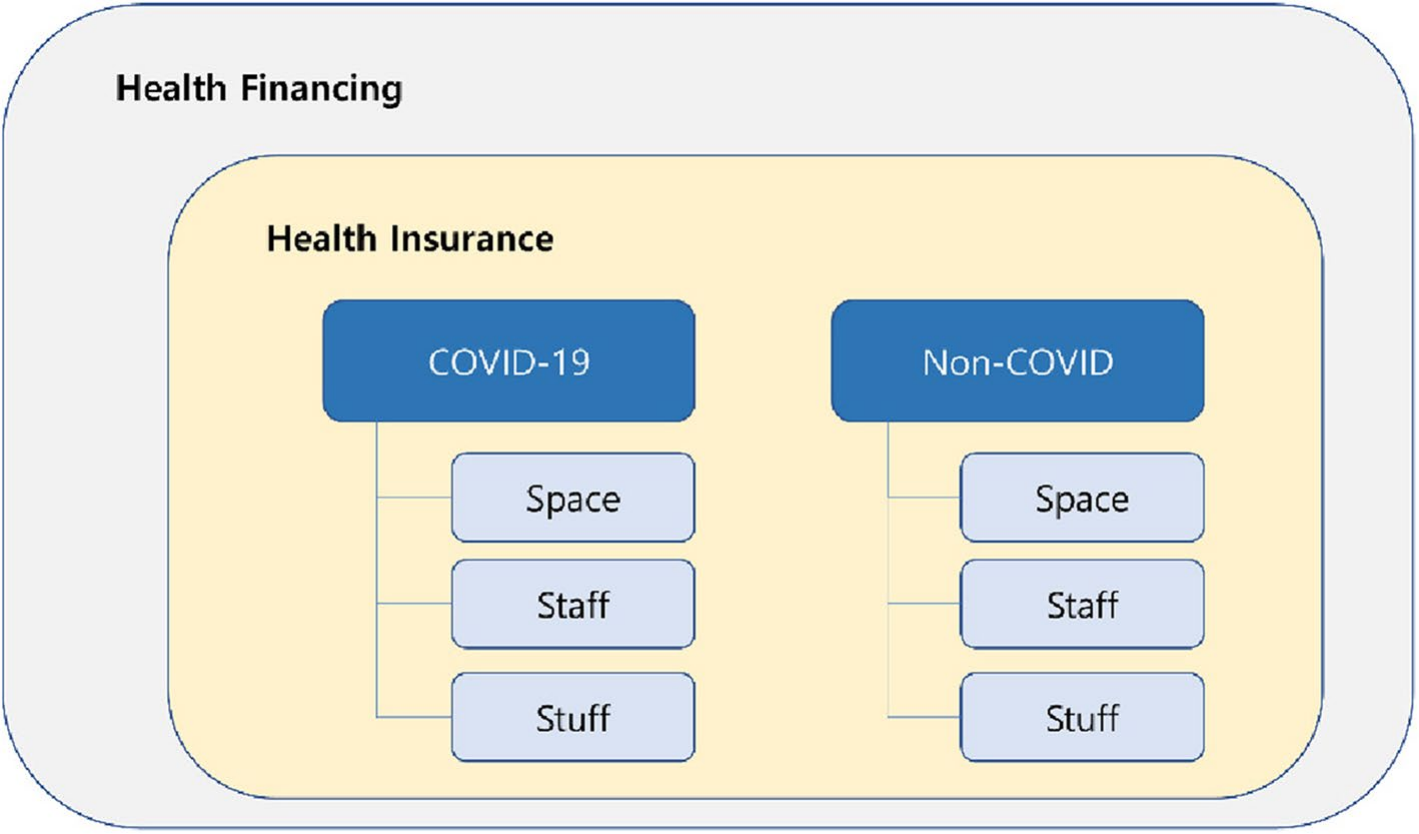
Analytical framework for comparing COVID-19 health insurance response

We developed an analytical framework for the analysis of domains in which financial support (in the form of health insurance) was provided in response to COVID-19 (Fig. 1). The classification of different response policies for each analyzed country based on this framework ensured the clear identification of the response domains. The analysis was conducted in the following stages: data collection, classification of response domains and timeline, detailed support methods, and comparison by nation.

First, the classification involved health service domains dedicated to treating COVID-19 patients (COVID-19) and non-COVID-19 patients (non-COVID-19) (main domain). Treatments for COVID-19 fell under the “COVID-19” domain. Other treatments were classified under the “non-COVID-19” (hereafter “non-COVID”) domain. Characteristically for an infectious disease, the provision of health services in response to COVID-19 requires a dual-track health system that involves not only treatment and infection prevention but also the recovery of the health service delivery system for other patients [5]. That is, the provision of prevention-diagnosis-quarantine-treatment services and the management of accumulated health care demand arising from the response to COVID-19 must occur simultaneously. Thus, the “COVID-19” domain identified policies to support the expansion of the health service providers’ capabilities in response to the rapid surge in demand caused by COVID-19. However, the “non-COVID” domain identified the policies supporting the maintenance of existing health service functions and minimizing the impact of the COVID-19 response on pre-existing services.

The main domain was divided into sub-domains based on surge capacity (i.e., the capacity to improve service provision through the management of resources in response to a medical surge) [23, 24]. Even in situations with unexpected surges in health service needs, health services must be provided to as many individuals as possible within the population [25], and the services must be affordable and available to ensure access, which must also be supported with sufficient capacity [26]. Accordingly, we classified what incentives were offered to maximize surge capacity through health insurance. The main strategies proposed to boost and optimize the healthcare system’s response capacity include mobilizing staff, boosting supplies, and optimizing space [26–28]. Thus, the sub-domains were space, staff, and stuff. Space included financial support to provide the basic space required for health service provision (e.g., hospital beds). In other words, it referred to the expenses associated with the locations where healthcare services were provided, covering the universal and fundamental patient care provided at those locations. Staff included incentives offered for health services provided by the medical staff. This pertained to the healthcare services offered to patients based on medical judgments considering factors such as the severity of the patients’ conditions, their health status, and their individual needs. Stuff included additional compensation for medical equipment, treatment materials, and pharmaceuticals required to provide health services.

### Overview of target countries

Socio-economic status, COVID-19 status, existing health resources, and health insurance system were identified in the four analyzed countries (Table 1).

**Table 1:** Overview of the analyzed countries

Classification			Republic of Korea	Japan	Germany	United States of America
**Socio-economic status**	**Gross Domestic Product (GDP) (Current prices, current PPPs)** (USD, billion)^a^		2,344.3	5,315.6	4,560.9	20,893.7
	**GDP per head (Current prices, current PPPs)** (US Dollar)^a^		45,274	42,285	54,844	63,285
	**Current expenditure on health (all functions)** (share of GDP)^b^		8.4	11.1	12.8	18.8
**Status of COVID-19**	**Total population** (1,000 persons)^c^		51,269	126,476	83,784	331,003
	**Number of confirmed cases** (persons)^d^		56,872	217,312	1,640,858	18,648,989
	**Cumulative number of confirmed cases** (per million)^d^		1,109	1,718	19,584	56,341
	**Cumulative number of deaths** (per million)^**d**^		16	25	355	991
	**Date of first confirmed case**^**d**^		2020.1.20	2020.1.15	2020.1.28	2020.1.31
**Status of health resources**^e^	**Number of physicians per 1,000 persons**		2.4	2.5	4.3	2.6
	**Number of nurses per 1,000 persons**		7.2	11.8	13.0	11.9
	**Number of hospital beds per 1,000 persons**		12.4	13.0	8.0	2.8
	**Number of public hospital beds per 1,000 persons**		1.2	3.5	3.2	0.6
**Type of Healthcare system**^f^			Social Health Insurance	Social Health Insurance	Social Health Insurance	Private Health System (Partially social health insurance)
**Characteristics of social health insurance**	**Population coverage**^g^		97%	100%	87%	18%
	**Reimbursement system**	**Outpatient**	FFS	FFS	FFS under sectoral budgeting	FFS
		**Inpatient**	FFS (partially DRG)	DPC	DRG	DRG

*PPP* Purchasing power parity, *FFS* Fee-for-services, *DRG* Diagnosis-related group, *DPC* Diagnosis-procedure combination

^a^2020 statistics [29]

^b^2020 statistics [30]

^c^2019 statistics [31]

^d^Statistics as of December, 2020 [32]

^e^2018 statistics [30]

^f^ [33]

^g^ [34–37]

In this study, the four countries are Organisation for Economic Co-operation and Development (OECD) members, with the United States having the largest gross domestic product (GDP) among OECD countries (2020 statistics); Japan, Germany, and South Korea ranked second, third, and eighth, respectively. All four countries have a GDP per capita(current prices, current PPP, 2020 statistics) of more than USD 40,000, which is higher than the OECD average of USD 45,025 for the three countries except Japan [29].

In Korea, the 3 T strategy (pre-emptive testing, prompt tracing, and proper treatment) was used to effectively reduce the incidence rate of confirmed cases in the early stages of the COVID-19 pandemic [38]. The Korean health insurance system covers 97% of Koreans, in a single insurer system [36].

Japan experienced a surge of confirmed cases in major cities, beginning with small mass infections, but was criticized in the initial response stage for the insufficient availability of polymerase chain reaction (PCR) tests and the lack of cooperative efforts between government and medical institutions [39, 40]. The nationwide health insurance program was established in 1961, after which all Japanese citizens were eligible for health insurance. Depending on the beneficiary characteristics, employee health insurance and community-based health insurance were implemented [41].

Germany has been able to reduce its COVID-19 mortality rate presumably due to the relative abundance of its resources, including beds and care providers, and its nationwide health insurance system has enabled the coverage of treatment and diagnostic costs [42]. Approximately 87% of Germans are enrolled in the national public health insurance system, which individuals who earn less than a certain income are obliged to subscribe to [34]. Contributions are structured so that the collected contributions of the health insurance association are consolidated into the health fund and distributed to the health insurance association [43].

The United States has the highest cumulative number of confirmed cases in the world. The American health insurance system is operated under a private health insurance market based on voluntary enrolment along with Medicare—a public health insurance system that provides health services for adults aged 65 years or older and those with a disability—as well as Medicaid—a public welfare system for low-income individuals. This study examined the policy intervention of Medicare, in which approximately 18.6% of Americans are enrolled [44].

Since private health insurance is at the center of the American health insurance system, the number of public beds per population is among the lowest among OECD countries. As the United States primarily relies on private health insurance, its health system differs from that of the other three countries. However, the relatively low number of public hospital beds resulting in most healthcare services delivered by private providers is similar to the situation in Korea [45]. Additionally, the Medicare program wields significant influence over the American healthcare system. The Center for Medicare and Medicaid Innovation (Innovation Center) devises and tests new Medicare policies considering the synergy with the private sector [46]. In fact, the new regulations and incentives introduced by Medicare policies significantly influence provider behavior, affecting not only Medicare patients but also non-Medicare patients [47]. As we aimed to identify effective incentive-based strategies for providers in response to surge capacity needs, we included both in the analysis.

### Data collection

We collected data from the official websites of each nation’s Ministry of Health and social health insurers. Since data could be quickly and reliably obtained from this source in the changing pandemic situation, data collection through websites was carried out first. For comprehensive data collection, additional relevant contents and data were also collected from the OECD and the European Observatory on Health Systems and Policies websites, which offer up-to-date compilations and disclosures of COVID-19-related health service policies (see Additional file 1).

We searched for data using keywords related to our topic. The keywords included “COVID-19,” “coronavirus,” “health system,” “health policy,” and “health insurance.” “Medicare” was added to the search in the American context. The collected data included publications from the European Observatory on Health Systems and Policies; publications, web pages, and press releases from governments or health insurers; and an OECD research report (see Additional file 2). The collected data were primarily in English, but data from each nation’s Ministry of Health websites were collected in Korean, Japanese, and German languages, respectively. The collected data were primarily based on title and abstract screening to confirm the relevance to the research topic. Only the materials deemed appropriate for the analytical framework of this study were selected, and data unrelated to COVID-19 and health insurance were excluded. We reviewed the contents of the screened articles, and articles meeting the following conditions were excluded from the analysis: 1) support policies that used government financing other than health insurance resources, 2) policy implementation period outside of 2020, and 3) duplicate contents in multiple articles. Articles on changes in administrative regulations related to cost reimbursement for medical institutions were reviewed by considering them as indirect financial support policies even if there was no additional health insurance financial support. Consequently, a total of 16 articles were analyzed.

The final data were analyzed and classified according to the main domains and sub-domains. The data were classified into the corresponding domains after verification and clarification of the data analysis process based on consensus in a meeting of researchers. That is, in case of disagreement on classification among researchers, consensus was reached on whether it was appropriate to classify data under a specific domain via discussion.

## Results

### COVID-19 wave and timeline of health insurance response

**Fig. 2:**
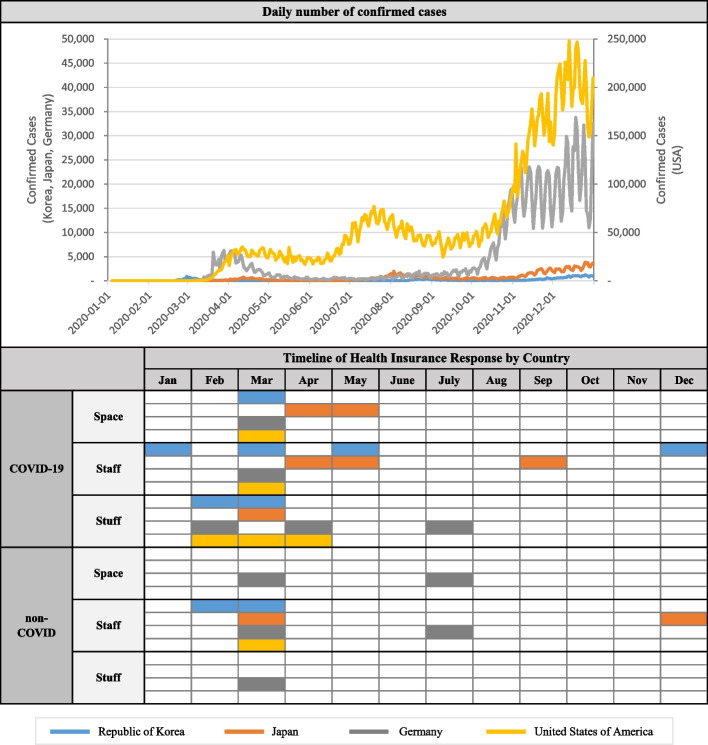
Timeline of Policy Implementation by Country

Prior to comparing the financial support policies regarding the COVID-19 pandemic, the current COVID-19 status of each country was examined (Fig. 2). The four countries shared a similar pattern in which the first confirmed case of COVID-19 was reported in January 2020 and increases in confirmed case numbers occurred around March to April. Subsequently, a slight decrease was observed in case numbers until the case numbers increased once again at various times in the three countries, excluding Germany (Korea – September, Japan – August, United States – July–August). In November, another wave in confirmed case numbers was observed.

Examining the pattern of COVID-19 outbreaks and the timing of health insurance intervention implementation reveals that a comprehensive policy implementation of space, staff, and stuff occurred for both the “COVID-19” and “non-COVID” domains in March and April, when the confirmed case numbers rapidly increased.

All four countries implemented simultaneous health insurance policy interventions during the first wave, indicating that social health insurance initiated a response from the early stages of the COVID-19 spread. Therefore, the timing of health insurance system intervention during this major crisis affecting the healthcare systems, namely, the first wave of COVID-19, seemed to be minimally influenced by factors such as the composition of private providers, population coverage, and characteristics of the reimbursement systems.

Among the systems, the implementation of staff was quickest in Korea’s COVID-19 response, as were its responses in the “non-COVID” domain. The single-payer and fee-for-service (FFS) framework of Korea’s incentive system facilitated a relatively smooth increase in testing capacity by healthcare professionals within a short period.

Even after the initial policy intervention, continuous policy intervention efforts, including the supplementation of existing interventions or implementation of additional policies, were observed regardless of the increase pattern of confirmed cases. This trend appeared consistent across all countries, irrespective of the characteristics of their respective health systems. However, the United States concentrated its main support efforts in the COVID-19 domain, with the timing of its interventions tending to align with the first wave. Its degree of health insurance intervention might have been lower than that in the other countries because its Medicare covers a limited population compared with the national insurance in the other countries.

### Health insurance response according to analytical framework

We compared the detailed response domains and health insurance implementation targeting COVID-19 and non-COVID patients in each country based on our analytical framework (Table 2). Generally, the COVID-19 domain commonly involved additional compensation to address costs related to providing inpatient services for confirmed patients. Given the need for inpatient treatment for highly contagious confirmed COVID-19 patients using negative pressure wards to prevent transmission to other patients, costs pertaining to such services were made eligible to claim. The space sub-domain involved compensation for the hospitalization fees of COVID-19 patients—the most basic of inpatient service costs. In both Korea and the United States, the hospitalization fees for confirmed patients were increased by 20% compared to the existing level of compensation for inpatient care fees [9, 48]. In Japan, an additional fee for inpatient care for type-2 infectious disease is charged from patients with an infectious disease to account for the increase (up to four times) in infection risk [49]. In the United States, hospitalization fees were increased in accordance with the Coronavirus Aid, Relief, and Economic Security Act (hereafter CARES Act), applied retroactively from January 27, before the enforcement date.

**Table 2:** Health insurance responses to COVID-19 by country

Classification		Republic of Korea						Japan					Germany				United States of America			
		Inpatient/Outpatient	Content(s)			Health insurance fee (USD^a^)	Month Introduced	Inpatient/Outpatient	Content(s)		Health insurance fee (USD^a^)	Month Introduced	Inpatient/ Outpatient	Content(s)	Health insurance fee (USD^a^)	Month Introduced	Inpatient/Outpatient	Content(s)	Health insurance fee (USD^a^)	Month Introduced
**COVID-19**	**Space**	Inpatient	Negative pressure isolation room inpatient care fee			341.95	Mar	Inpatient	ICU inpatient care fee (admission within 3 days)		1,424.21	Apr	Inpatient	Fee for additional ICU bed with ventilation	56,818.18/bed	Mar	Inpatient	Inpatient care fee (confirmed patient)	20% increase compared to existing rate	Mar
											2,136.32	May								
			ICU inpatient care fee (general, wards dedicated to severe COVID-19 patients)			99.00	Mar		Additional fee for inpatient care with type-2 infectious disease		21.93	Apr								
											87.72									
	**Staff**	Inpatient	Negative pressure isolation management fee in ICU	General		215.62	Mar	Outpatient	Dental consultation fee for confirmed patient		26.14	Apr	Inpatient	Additional daily nursing fee	43.18/day	Mar				
				Wards dedicated to severe COVID-19 patients		431.25														
			Inpatient isolation management fees in long-term care hospitals			72.75	May													
			Infection prevention and control fee			2.86	Jan	Inpatient	Additional fees for emergency management		166.67	Apr								
			Residential treatment center patient care fee		I	31.43	Mar				250	May								
						47.15	Dec													
					II	20.97	Mar													
						31.46	Dec													
					III	94.3	Dec													
			Hemodialysis fee			270.68	Dec				416.67	Sep								
	**Stuff**	Inpatient and outpatient	PCR test			64.85	Feb	Inpatient and outpatient	PCR test		118.42	Mar	Inpatient and outpatient	PCR test	67.05	Feb				
		Inpatient	Mask for medical staff (negative pressure isolation room, isolation room in ICU)			19.87	Mar						Inpatient	PPE costs	56.82/patient	Apr	Inpatient and outpatient	PCR test	26.00	Feb
															113.64/patient	Jul			51.00	Mar
																			100.00	Apr
**Non-COVID**	**Space**	-						-					Inpatient	Compensation for empty beds	636.36/bed	Mar	-			
	**Staff**	Outpatient	Telephone consultation fee (clinic-level)			4.16	Feb	Outpatient	Telephone consultation fee (first visit)		18.77	Dec					Outpatient	Expansion of telemedicine coverage	Same as previous	Mar
		Outpatient	Authorization of proxies (prescription drugs) (first visit, clinic-level)			4.96	Feb		Additional fee for consultation (under 6 years old)		8.77	Apr								
		Outpatient	Infection prevention and control fee (clinics dedicated to respiratory diseases)			17.63	Feb		Infection prevention and control fee (suspected patient)		26.32	Apr								
		Inpatient and outpatient	Transmission prevention management fees in national safe hospitals			17.63	Feb		Additional examination fee for consultation	Outpatient	0.44	Dec			409.09 ~ 863.64/bed	Jul				
										Prescription	0.35	Dec								
										Visiting nurse	0.44	Dec								
		Inpatient	Infection prevention and control fee (mental hospital, long-term care hospital)			0.99/day	Apr	Inpatient		Admission	0.88	Dec								
	**Stuff**	-						-									-			

^a^USD 1=KRW 1,187=JPY 114=EUR 0.88

*ICU* Intensive care unit

As the compensation of hospitalization fees in the United States was based on the diagnosis-related group (hereafter DRG), the increase in fees offered comprehensive support for not only space but also staff and stuff. The hospitalization fees for intensive care units (ICUs) were increased by 6% in Korea [9] while, in Japan, two revisions led to a fourfold increase in fees [50]. Such increases in hospitalization fees were made considering the additional need for care providers to treat severe COVID-19 patients who require more extensive management (including equipment such as an extracorporeal membrane oxygenation machines and ventilators) as well as the reduced efficiency of care due to personal protective equipment (PPE). There were no indications of additional increases in existing hospital fees in Germany, but support was provided for the costs involved in gaining access to hospital beds [15]. The amount of support provided per ICU bed equipped with negative-pressure facilities was equivalent to USD 56,818.18 (EUR 50,000) per bed.

The staff sub-domain involved support similar to that observed in the space sub-domain. This includes compensation for the burden placed on health care practitioners who care for confirmed patients receiving inpatient treatment. In Japan, support was also provided for outpatient services.

A detailed examination of benefits related to hospitalization fees revealed an increase in hospitalization fees in the space sub-domain, concurrent with inpatient management costs in the staff sub-domain [9]. First, the negative pressure isolation management fee in ICUs had doubled to USD 215.62 (KRW 255,940) while the negative pressure isolation management fee in ICU wards dedicated to severe COVID-19 patients had tripled to USD 431.25 (KRW 511,890). Furthermore, an infection prevention and control fee was newly established (USD 2.86, KRW 3,390) given that COVID-19 patients require isolation and additional efforts to prevent transmission to other patients. Similarly, an additional fee for emergency management was implemented in Japan [49]. Considering the potential additional treatments and tests needed to identify patient statistics as well as ordering additional drugs and ventilators in the process of caring for critically ill confirmed patients, fees had increased by up to five times. In Germany, compensation of USD 43.18 (EUR 38) was also provided for the increase in nursing work for treating confirmed patients [15].

Moreover, due to the difficulties in securing hospital beds in Korea, “residential treatment centers” were created for confirmed COVID-19 patients with mild symptoms for monitoring, diagnosing, and testing and for transferring worsening cases to nearby hospitals. Based on the type of service provided in each facility, costs were classified as I, II, or III and compensated accordingly [9]. Category I included support for patient consultations, monitoring, and chest X-rays through the provision of healthcare practitioners and X-ray equipment. Category II included the same services as under Category I (excluding X-rays), and Category III included greater benefits for patients admitted to one of the main residential treatment centers equipped with better medical facilities or those transferred to a residential treatment center from a medical institution as they required more intensive medical care than under Category I or II.

Among confirmed patients, those requiring specific care received relevant benefits. In Korea, compensations of USD 270.68 (KRW 321,300) were provided for treating dialysis patients [9] while, in Japan, compensations of USD 26.14 (JPY 2,980) were provided for outpatient dental treatment [51].

The stuff sub-domain involved compensations to alleviate the economic burden of PPE such as masks. Among the countries analyzed, compensation for PPE was provided in Korea and Germany. In Germany, compensation for PPE was increased from the initial amount of USD 56.82 (EUR 50) in April to USD 113.64 (EUR 100) in July [15]. Furthermore, compensation was also provided for PCR tests used to confirm COVID-19 infection. This benefit was applied in Korea, Germany, and the United States in February and in Japan in March, more quickly than benefits related to the treatment of confirmed patients [9, 52–55]. Furthermore, in February, the United States first approved a testing kit developed by the Centers for Disease Control and Prevention to confirm COVID-19 diagnoses and extended its insurance benefits to include the usage of such testing kits developed in the private sector to expand supply levels. In April, the insured value for testing kits increased from USD 51 to USD 100 [53, 54]. Germany also approved testing kits in February and introduced an insured value of USD 67.05 (EUR 59) [26].

The non-COVID domain mainly involved policy interventions in the staff sub-domain. No policy interventions were implemented for the space and stuff sub-domains other than the comprehensive benefits in Germany provided for the reduction in patients due to COVID-19**.** In Germany, an insurance fee of USD 409.09–863.64 (EURO 360–760) was applied to account for the reduction in patients resulting from postponed treatments or delays due to the treatment of COVID-19 patients [15].

While support in the COVID-19 domain was focused on inpatient services, support in the non-COVID domain usually involved support for outpatient services. In the staff sub-domain, all countries except Germany expanded benefits for virtual consultations. Telemedicine was implemented in Korea and Japan, where virtual consultations were previously not permitted, for a cost of USD 4.16 (KRW 4,940) and USD 18.77 (JPY 2,140), respectively [50]. In Korea, the respective fee could be claimed in addition to the basic consultation fee while, in Japan, USD 18.77 (JPY 2,140)—an amount lower than the previous initial consultation fee of USD 25.26 (JPY 2,880)—could be claimed. In the United States, where virtual consultations had already been available, only fees occurring in rural areas or areas with a shortage of healthcare practitioners were covered. Meanwhile, during the public health emergency period due to COVID-19, compensations were made eligible for a greater scope of services, locations, and service methods (e.g., video, telephone, online) [56]. Furthermore, in Korea, families of patients with a chronic disease requiring long-term prescriptions were granted permission for proxy access to prescriptions with an insurance fee of USD 4.96 (KRW 5,890), approximately 50% of the existing consultation fees.

Furthermore, to create a safe environment for non-COVID patients to receive treatment while preventing infections, compensation was also implemented for such services. In Korea, National Safe Hospitals and clinics dedicated to respiratory diseases, with segregated access, treatment, and care settings, were introduced to enable non-COVID patients to receive in-person treatments without the fear of infection, along with an infection prevention and control fee (USD 17.63, KRW 20,930). An infection prevention and control fee (USD 0.99, KRW 1,170) for implementing infection control protocols such as mask-wearing was also provided for patients in locked wards in mental and long-term care hospitals with many vulnerable elderly patients [9].

However, in Japan, compensations were offered for all outpatient or inpatient services, drug dispensing, and visiting nursing care without additional conditions [57]. Furthermore, for the testing of untested suspected COVID-19 patients, the infection prevention and control fee of USD 26.32 (JPY 3,000) was compensated [49]. Additional benefits included compensations for children under the age of 6 considering the higher likelihood of contact with healthcare practitioners [51].

Such policy support for health insurance was made possible through health insurance financing as well as national financial support. In Japan, the government budget regularly contributed toward health insurance financing as various support measures were modified or added [51, 58]. In Germany, the Hospital Relief Act was enacted in March 2020 to alleviate the financial support and administrative burden of medical institutions through the various aforementioned health insurance policies. Additional funding for this implementation was provided through taxes and contributions [59]. In the United States, in accordance with the CARES Act enacted in March 2020, matters related to the expansion of Medicare coverage and the like were stipulated along with an announcement that the funds for the changes would be sourced from the national disaster medical system [26].

### Easing administrative regulations of health insurance

Aside from the additional financial compensation, regulations pertaining to health insurance administration procedures were relaxed to increase efficiency in the management of medical institutions (Table 3).

**Table 3:** Changes in administrative regulatory policies for health insurance

Republic of Korea	Japan	Germany	United States of America
• Advance reimbursement before utilization review • Suspension of reporting institutional changes (staff, facilities) • Exclusion from bundled payment on COVID-19-related medical services	• Exception of reimbursement reduction related to additional beds when the approved number of beds under the Medical Act is exceeded • Suspension of reporting institutional changes (staff, facilities)	• Exception for the review of COVID-19-related claims • Shortening reimbursement period	• Waiving utilization review during public health emergency period

In Germany and the United States, the screening of medical expenses during a COVID-19 outbreak, or for COVID-19-related activities, was eased [59, 60]. Such measures were enforced in recognition that services were provided based on medical necessity without evaluating the adequacy of the provided medical service to alleviate the time and cost needed for the review process considering the situation’s urgency [59].

In addition to the easing of the review of medical expenses, the reimbursement period of health insurance claims was shortened in Korea and Germany [15, 61]. In Korea, payments were made in advance (90–100% of health insurance benefits for the same month of the previous year paid to the medical institution prior to settlement) or early (90% of the payment is made 10 days after the claim and before the completion of the evaluation). Through this process, the usual period of 22 days that it took medical institutions to receive the payment was reduced to 10 days.

Existing obligations to report staff or facility changes in institutions were also suspended. Although, in Korea, changes in human resource are linked to differential compensation payments and thus must be updated promptly, the duty to report was suspended. Additionally, contents of previous reports were applied in response to the frequent occurrence of unavoidable changes such as the isolation of healthcare practitioners after close contact, treatment of confirmed COVID-19 patients, or dispatch to other medical institutions [61]. In Japan, although a reduction in medical expenses is applied if the approved number of beds exceeds the number outlined under the Medical Act, the reduction was not applied for beds used to treat COVID-19 patients [50]. Furthermore, similar to Korea, reports on the changes in human resources due to a temporary surge in patients or the isolation of medical staff were suspended. Moreover, in the context of Korea, although DRG payment is underway for certain diseases or institutions, compensations of COVID-19 patients were based on fee-for-service payment to prevent the likelihood of under-compensation that can occur with bundled payments [9].

## Discussion

This study revealed that the analyzed countries provided financial support through health insurance from the initial stage of the pandemic. Although there are differences across countries in the detailed response and amount of support, all countries shared a common purpose of maintaining or expanding service providers’ capacity to provide services throughout the pandemic. Comprehensive policy interventions were implemented in both the COVID-19 and non-COVID domains. Among these, support in the COVID-19 domain involved space, staff, and stuff sub-domains while support in the non-COVID domain focused primarily on the staff sub-domain. The support across the three sub-domains can be summarized as follows:

First, financial support in space and staff both served to alleviate the increased workload burden that arose from changes in the service environment. Expansions and changes occurred in the space sub-domain due to the shortage of facilities relative to the number of COVID-19 patients. Aside from COVID-19, pandemics such as the 2009 H1N1 virus outbreak also led to increased healthcare demands due to hospitalizations. During that time, similar to the COVID-19 pandemic, attempts to expand service capacity, such as converting alternative spaces to hospital beds, were employed as strategies [62]. Thus, changes occurred in healthcare practitioners’ line of service and the management of inpatient services. Additionally, situations arose in which locations not originally used to deliver health services were used due to the increased need for negative pressure isolation rooms given the characteristics of infectious diseases. In these cases, compensation for inpatient services was increased from 20 to 400% of the previous costs to accommodate changes in the workload of healthcare practitioners.

Support for increased workload was also implemented in the staff sub-domain. Compared to the space sub-domain, in which support was focused primarily on inpatient services, support in the staff sub-domain comprised more comprehensive interventions including outpatient services. In the COVID-19 domain, support for infection prevention and control among hospitalized patients was provided in Korea while, in Germany, nurses’ compensation was increased. Moreover, compensation for specialized services, such as dental treatments and hemodialysis, was also reinforced. In the non-COVID domain, additional compensations were made toward healthcare practitioners’ protection during interactions with patients—both in general and those suspected of having COVID-19. In Japan, considerations were also given to additional work involving pediatric care.

The lack of physical space due to increased patient numbers was foreseen, but the staff sub-domain emerged as the most limited domain throughout the pandemic [63]. Previous research has also demonstrated that, although the service capacity of each country may vary, investments are needed to improve the quantity and quality of healthcare practitioners accompanied by access to sufficient numbers, education, and willingness of practitioners [13]. During a pandemic, more staff are required than usual for the same type of service due to the need for staff to wear PPE. Thus, support included a comprehensive consideration of the increased workload and the mental and physical burden placed on healthcare practitioners.

Second, financial support in the staff sub-domain was reflected in changes in the healthcare delivery system. Quarantining the sick, contact tracing, and social distancing were employed globally to prevent transmission during the early stages of COVID-19 [14]. These strategies have also been adopted as measures to ensure effective delivery of health services, and health insurance supported these measures comprehensively.

To prevent the transmission of COVID-19, notable changes in the delivery of outpatient services occurred in the non-COVID domain. First, compensations for virtual consultations, such as telemedicine, were reinforced in the analyzed countries (except Germany). In the case of the United States, although telemedicine was previously authorized, the scope of its application was initially limited. During the COVID-19 pandemic, however, the geographical requirements and scope of application for telemedicine services were expanded, and the level of support was maintained. In Korea and Japan, telemedicine was not previously established, but methods such as telephone consultations and prescription proxies were actively implemented. Such actions helped compensate for the reduced number of patients and service provisions due to COVID-19.

Furthermore, respiratory and non-respiratory patients were instructed to use separate healthcare access and treatment paths in Korea in addition to the establishment of National Safe Hospitals and clinics dedicated to respiratory disease for the safe provision of services. Such institutions were allocated new functions and roles in addition to financial support for the additional work required for patient management and service provision. In Korea, additional compensation was provided for health services in newly established residential treatment centers specifically for COVID-19 patients.

Non-COVID domains have also been threatened by the provision of COVID-19-related services throughout the pandemic [7, 8]. Thus, patients were managed to prevent the spread of infection from COVID patients to non-COVID patients and ensure a safe treatment environment. Healthcare professionals’ infection prevention efforts were supported by health insurance.

Third, the stuff sub-domain was focused on prevention of infection among healthcare professionals and patients. PPE, such as masks and protective clothing, worn by healthcare practitioners to prevent infection was compensated for. PPE was required for practitioners when interacting with patients, and, considering the additional costs involved, concerns were raised that a lack of financial support would lead to problems such as the inadequate use of PPE or passivity in treating patients.

Furthermore, extensive support was provided for the use of testing kits. Given the importance of increased testing at the initial stage to contain infections and preemptively treat confirmed cases, expanding diagnostic capacity for quick screening was an important factor of the national public health emergency policy for responding to COVID-19 [26, 64].

Fourth, support for healthcare services through health insurance enabled rapid response in the pandemic context and required government budget support. Insurance policies prompted healthcare providers to recognize COVID-19 and to swiftly implement changes to their patient care and patient care facilities. The associated costs to safeguard these providers were also compensated through these policies. Financial support in the form of increased fees for health services was facilitated using the existing insurance fee calculation system and payment process. Nevertheless, such financing required both health insurance financing and the government budget. Support through the government budget required approval from the federal government or the National Assembly to facilitate the smooth implementation of the national public health emergency policy and compensation for financial losses among healthcare practitioners; thus, the government’s role in health insurance financing also expanded during the pandemic [8]. As the domains of financial support provided through health insurance are determined based on the direction of national public health emergency policy, health insurance financing complements the government budget support rather than relying solely on insurance funds.

According to a recent survey by the NHI Service, Koreans perceive the NHI system as a social safety net that enables access to care without the excessive burden of cost, despite the ongoing COVID-19 crisis [65]. Thus, it is evident that Koreans regard the NHI system positively and that the health insurance system is highly accepted by its users. Furthermore, support through health insurance has been regarded as one of the success factors of preventive measures against a widespread outbreak of COVID-19 [66], indicating a need for government support for health insurance policies during this pandemic.

### Limitations

This study examined support systems for health service providers in four countries operating social health insurance systems. The study limitations are as follows. Since the spread and severity of COVID-19 vary by country, there are differences in the direction of national public health emergency policy. From a health insurance perspective, however, an emphasis was placed on comparison, and, thus, such national specificities were not considered in detail. Furthermore, due to the varying financial conditions of each country, the financial capacity for health insurance may also differ, influencing policy interventions. Another limitation was that only changes in the initial year of the pandemic were analyzed, thus failing to incorporate the ongoing, long-term policy changes. Nevertheless, this study aimed to derive the common trends in financial support between countries by identifying the specific domains of support in each country.

## Conclusions

To overcome the pandemic crisis, policymakers must consider not only quarantine policies but also health service support. Capacity expansion of health systems during the COVID-19 pandemic is a key factor in ensuring health system resilience [13]. In the COVID-19 pandemic, financial support through health insurance was provided at a higher than usual level with an emphasis on staff in both the COVID-19 and non-COVID domains. Such support enabled health service providers to continue to provide services without concerns regarding reduced profits or additional costs. This allowed for prompt and comprehensive capacity expansion and timely implementation of changed infection control policies.

Based on the lessons learned from COVID-19, decisions regarding health insurance policies in future pandemics should consider the following. First, it is important to recognize the changes that occur in service provision and the corresponding work-related burdens and to provide prompt additional financial support. Second, considering the consistency and relevance to the national public health emergency policy, support should be provided for health insurance plans that align with the goals and direction of the policy. Third, to enable access to sufficient financing in which appropriate financial support can be provided during a pandemic, it is necessary to monitor the financial sustainability of the health insurance system and establish a foundation for receiving governmental support. Fourth, continuous assessment of the response policies and their effects and financial requirements may help to establish the optimal health insurance response system to appropriately manage medical resources and operate a resilient health system.

### Supplementary Information

**Additional file 1.** Flow Chart of Literature Selection. Figure describing the process of identification, exclusion, and inclusion of relevant data sources.

**Additional file 2.** Summary of the Reviewed Literature. Table describing the collected data, including publications from the European Observatory on Health Systems and Policies; publications, web pages, and press releases from governments or health insurers; and an OECD research report.

## Data Availability

All data used in this study are publicly available.

## Abbreviations

COVID-19: Coronavirus disease 2019
NHI: National Health Insurance
QCA: Qualitative comparative analysis
OECD: Organisation for Economic Co-operation and Development
GDP: Gross domestic product
PCR: Polymerase chain reaction
DRG: Diagnosis-related group
ICU: Intensive care unit
PPE: Personal protective equipment
