# Supplementary figures and images for: Lessons from health insurance responses in counteracting COVID-19: a qualitative comparative analysis of South Korea and three influential countries
Source: Arch Public Health. 2023 Nov 21;81:205. doi: 10.1186/s13690-023-01209-w (PMC10664685; doi:10.1186/s13690-023-01209-w)

Additional File 1: Flow Chart of Literature Selection


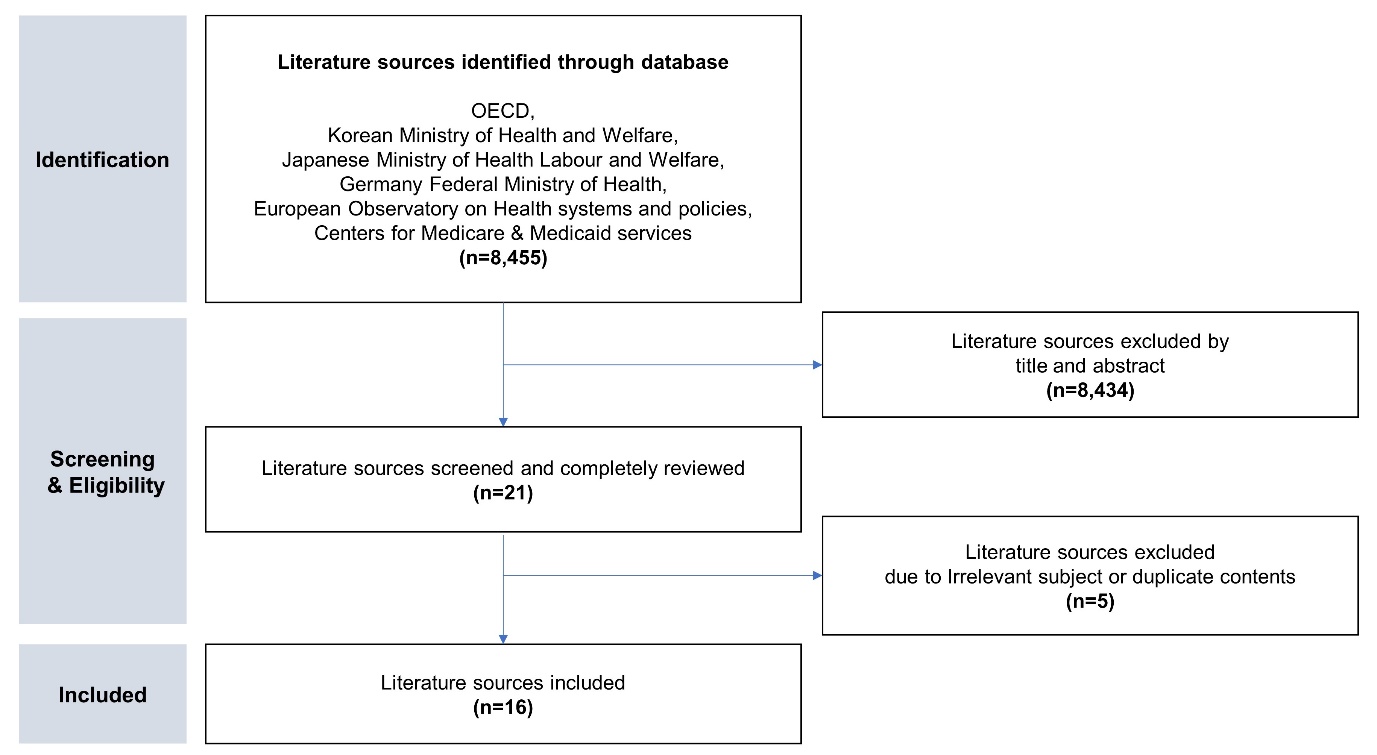

Supplement: Supplementary file 1 — Additional file 1. Flow Chart of Literature Selection. Figure describing the process of identification, exclusion, and inclusion of relevant data sources. [file 13690_2023_1209_MOESM1_ESM.docx]
