# Supplementary material for: Lessons from health insurance responses in counteracting COVID-19: a qualitative comparative analysis of South Korea and three influential countries
Source: Arch Public Health. 2023 Nov 21;81:205. doi: 10.1186/s13690-023-01209-w (PMC10664685; doi:10.1186/s13690-023-01209-w)
Supplement: Supplementary file 2 — Additional file 2. Summary of the Reviewed Literature. Table describing the collected data, including publications from the European Observatory on Health Systems and Policies; publications, web pages, and press releases from governments or health insurers; and an OECD research report. [file 13690_2023_1209_MOESM2_ESM.docx]

Additional File 2: Summary of the Reviewed Literature

| **Country** | **Category**  **(Key findings)** | **Sub-category** | **Type** | **Author and Institutions** | **Title** | **Language** | **Published**  **Year** |
| --- | --- | --- | --- | --- | --- | --- | --- |
| **Republic of Korea** | Health insurance financial responses | - | Government  document | Ministry of Health and Welfare | At a Glance: Updated Health Insurance Payments Related to COVID-19 | Korean | 2021 |
|  | Health insurance | - | Government  document | Ministry of Health and Welfare | Guidance for Support for Medical Institutions | Korean | 2020.5.27. |
| **Japan** | Health insurance financial responses | Inpatient, emergency services etc. | Webpage | Ministry of Health, Labour and Welfare | Special Response to Health Services Compensations Related to the Acceptance of COVID-19 Patients | Japanese | 2020.5.25. |
|  | Health insurance financial responses | Consultation fee for dental services, etc. | Webpage | Ministry of Health, Labour and Welfare | Additional Support for Medical Institutions Responding to COVID-19 | Japanese | - |
|  | Health insurance financial responses | Governmental funding | Webpage | Ministry of Health, Labour and Welfare | Additional Support for Medical Institutions Responding to COVID-19 | Japanese | - |
|  | Health insurance financial responses | PCR test | Webpage | Ministry of Health, Labour and Welfare | Response to COVID-19 | Japanese | 2021.7.7. |
|  | Health insurance financial responses & administrative process | Inpatient  & administrative process | Webpage | Ministry of Health, Labour and Welfare | Special Response to Health Services Compensations Related to the Acceptance of COVID-19 Patients | Japanese | 2020.9.14. |
| **Germany** | Health insurance financial responses | Additional beds, nursing fee, etc. | Article | Quentin W et al.  (Eurohealth) | Adjusting Hospital Inpatient Payment Systems for COVID-19 | English | 2020 |
|  | Health insurance financial responses | PCR test | Report | OECD | Beyond Containment: Health Systems Responses to COVID-19 in the OECD | English | 2020 |
|  | Health insurance financial responses & administrative process | Governmental funding  & administrative process | Webpage | European Observatory on Health Systems and Policies | Health Financing - COVID-19 Hospital Relief Act: Financial Support for Hospitals | English | 2020.11.9. |
| **United States of America** | Health insurance financial responses | Inpatient | Webpage | Centers for Medicare & Medicaid Services | July 2020 Quarterly Update to the Inpatient Prospective Payment System | English | 2020.4.24. |
|  | Health insurance financial responses | PCR test | Press release | Centers for Medicare & Medicaid Services | CMS Changes Medicare Payment to Support Faster COVID-19 Diagnostic Testing | English | 2020.10.15. |
|  | Health insurance financial responses | PCR test | Press release | Centers for Medicare & Medicaid Services | CMS Develops Additional Code for Coronavirus Lab Tests | English | 2020.3.5. |
|  | Health insurance financial responses | Telemedicine | Webpage | Centers for Medicare & Medicaid Services | Medicare Telemedicine Health Care Provider Fact Sheet | English | 2020.3.17. |
|  | Health insurance financial responses | Governmental funding | Report | OECD | Beyond Containment: Health Systems Responses to COVID-19 in the OECD | English | 2020 |
|  | Health insurance administrative process | - | Webpage | Centers for Medicare & Medicaid Services | COVID-19 Emergency Declaration Blanket Waivers for Health Care Providers | English | 2021.11.29. |

※ CMS: Centers for Medicare & Medicaid Services
